# p16 controls epithelial cell growth and suppresses carcinogenesis through mechanisms that do not require RB1 function

**DOI:** 10.1038/oncsis.2017.5

**Published:** 2017-04-17

**Authors:** M Sen, N Akeno, A Reece, A L Miller, D S Simpson, K A Wikenheiser-Brokamp

**Affiliations:** 1 Division of Pathology & Laboratory Medicine, Cincinnati Children’s Hospital Medical Center, Cincinnati, OH, USA; 2 Perinatal Institute, Division of Pulmonary Biology, Cincinnati Children’s Hospital Medical Center, Cincinnati, OH, USA; 3 Department of Pathology & Laboratory Medicine, University of Cincinnati College of Medicine, Cincinnati, OH, USA

## Abstract

The p16/RB1 tumor suppressor pathway is inactivated in the vast majority, if not all, human cancers. The current paradigm is that p16 and RB1 function in a linear pathway to suppress tumorigenesis; however p16 is preferentially lost in human cancers suggesting that p16 has critical tumor suppressive functions not mediated through RB1. Carcinomas arise from transformed epithelial cells and account for 80% of adult malignancies highlighting the need to understand p16/RB1 pathway function in organ epithelia. Lung cancer is the leading cause of cancer deaths and is associated with p16/RB1 pathway deregulation. We demonstrate that p16 is upregulated in the lung epithelium after *Rb1* ablation in genetically engineered mouse models. In contrast to fibroblasts, loss of RB1 family proteins, p107 or p130, did not result in p16 induction, demonstrating that p16 suppression is a unique RB1 pocket protein function in the lung epithelium *in vivo*. p16 upregulation did not induce cellular senescence but rather promoted survival of RB1-deficient lung epithelial progenitor cells. Mechanistic studies show that p16 protects RB1-deficient cells from DNA damage. Consequently, additional loss of p16 led to genetic instability and increased susceptibility to cellular immortalization and transformation. Mice with combined RB1/p16-deficient lungs developed lung tumors including aggressive metastatic lung cancers. These studies identify p16 loss as a molecular event that causes genetic instability and directly demonstrate that p16 protects against DNA damage in the absence of RB1 function providing an explanation for why p16 is preferentially targeted in human cancers.

## Introduction

The tumor suppressor proteins p16 and RB1 function in a common pathway that is deregulated in the vast majority of human cancers.^1, 2^ The current paradigm is that p16 and RB1 function in a linear pathway wherein p16 blocks cell cycle progression by maintaining RB1 in a functionally active state by inhibiting Cyclin D/Cyclin-dependent kinase 4/6 dependent phosphorylation.^3^ As p16 loss and *Rb1* mutations both lead to loss of RB1 function, it is unclear why p16 and *Rb1* are targeted in specific cancers and why p16 loss occurs much more frequently and in a wider variety of cancer types than does *Rb1* mutation. Deletion of the p16 locus is the most frequent copy number alteration across 12 commonly occurring cancers types, and p16 is among the genes most frequently silenced by methylation.^1^ In contrast, *Rb1* mutations are only frequently detected in retinoblastoma and small cell lung cancer (SCLC).^4, 5^ The markedly increased frequency of p16 as compared to RB1 loss in human cancers suggests that p16 has critical tumor suppressive functions that are not mediated through RB1.

The p16/RB1 tumor suppressor pathway is deregulated in virtually all lung cancers providing strong evidence that loss of p16/RB1 pathway function is required for lung carcinogenesis.^4, 6, 7, 8^ Lung cancer is the leading cause of cancer related deaths and has a dismal overall 5 year survival rate of <20%.^9^ Lung cancers are divided into non-small cell lung cancer (NSCLC) and small cell lung cancer (SCLC) with p16 loss being detected in up to 80% of NSCLC and biallelic *Rb1* loss being obligatory for development of SCLC.^4, 6, 7, 8^ Previous studies by us and others demonstrate that RB1 loss targeted to the murine lung epithelium results in neuroendocrine cell hyperplasia with additional Trp53 loss being sufficient for progression to SCLC, an aggressive neuroendocrine malignancy.^10, 11, 12^ These results in mouse models are in accordance with the obligatory loss of RB1 and TP53 in human SCLC providing evidence that genetic mechanisms underlying lung carcinogenesis are conserved between mice and humans.^4^ Despite the frequent loss of p16 in human NSCLC, however, p16 or RB1 loss alone or in combination with Trp53 in genetically engineered mice is not sufficient for development of NSCLC. We previously demonstrated that p16 is induced after RB1 ablation in lung epithelial progenitor cells, namely Club and type II cells, believed to serve as cells of origin for NSCLC.^11, 13, 14^ Increased p16 expression is also reported after knockdown of RB1 or its family members, p107 (RB1l1) or p130 (RB1l2), in human fibroblasts in culture as well as being a hallmark of human papilloma virus-driven cervical and head and neck cancers wherein RB1 family function is lost due to E7 viral oncoprotein expression.^15, 16, 17^ Induction of p16 promotes cellular senescence to limit tumorigenesis with maintenance of senescence believed to be heavily reliant on active hypophosphorylated RB1.^18^ However, RB1 loss in the thyroid induces cellular senescence with additional loss of p16 promoting tumor progression.^19^ These results suggest that p16 associated cellular senescence antagonizes RB1-deficient carcinogenesis and provide evidence that p16 has tumor suppressive functions that are not mediated through RB1. In the current study, genetically engineered mouse models were used to determine the regulation and biologic significance of p16 induction in RB1-deficient lung epithelial cells that give rise to lung cancer; a common epithelial derived malignancy. We demonstrate that p16 suppression in the lung epithelium *in vivo* is a unique RB1 function, differing from the shared p107 and p130 function in fibroblasts.^15^ We also show that unlike in murine and human fibroblasts, RB1 loss in lung epithelial progenitor cells is sufficient to enhance growth providing evidence that p16/RB1 pathway function is distinct in epithelial cells.^20, 21, 22^ Importantly, p16 induction after RB1 loss was not associated with cellular senescence but rather protected lung epithelial progenitor cells from DNA damage and development of aggressive lung cancers. Together these studies directly demonstrate that p16 has tumor suppressive functions that are not mediated through RB1 and are critical for protecting against carcinogenesis.

## Results

### p16 repression in lung epithelial cells is a unique RB1 pocket protein function

**Figure 1:**
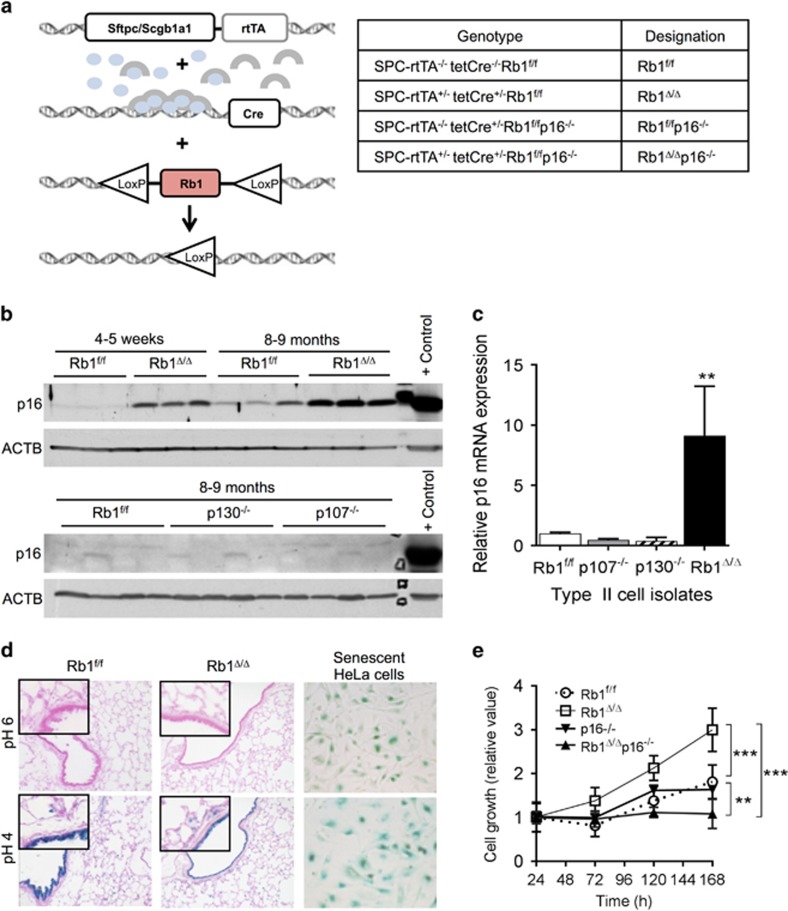
p16 suppression is a unique RB1 pocket protein function in the lung epithelium *in vivo* with p16 induction after RB1 loss functioning to enhance lung epithelial cell growth. (**a**) Sftpc/Scgb1a1-rtTA^+/−^; tetCre^+/−^ double transgenic mice were bred to Rb1^f/f^ and p16^−/−^ mice. Doxycycline (blue ovals) activates the rtTA (gray arches) leading to Cre-mediated Rb1 ablation in the lung epithelium. Genotypes resulting in the designated Rb1 and p16 status are indicated in the table. (**b**) p16 protein was induced in Rb1^Δ/Δ^ lungs from mice at 4–5 weeks of age with increased expression sustained in lungs from 8 to 9-month-old mice by western blot analysis. p16 protein was not induced in p107^−/−^ or p130^−/−^ lungs (*n*=4–9 mice per group). Lysates were evenly loaded as assessed by reprobing for β-Actin (ACTB). A 3T3 cellular lysate served as a positive p16 control. (**c**) p16 messenger RNA was induced in Rb1^Δ/Δ^, but not p107^−/−^ or p130^−/−^, type II cells as compared to Rb1^f/f^ controls by quantitative reverse transcription PCR (mean±s.d.; *n*=3–4 isolates per group; **P<0.01). (**d**) p16 induction in Rb1^Δ/Δ^ lungs was not associated with cellular senescence as assessed by senescence-associated β-galactosidase (SA-β-gal) activity at pH 6. Positive controls include SA-β-gal activity in senescent HeLa cells and lysosomal β-gal staining at pH 4 (*n*=3–4 mice per group at 9 months of age). Original magnifications: × 20 and insets × 40. (**e**) Growth of Rb1^Δ/Δ^ primary type II cells in culture was increased as compared to Rb1^f/f^ controls with additional loss of p16 reducing Rb1^Δ/Δ^ type II cell growth to below Rb1^f/f^ control levels. p16^−/−^ type II cell growth was similar to Rb1/p16-proficient Rb1^f/f^ controls (mean±s.d.; *n*=5–7 wells from 2 to 3 independent cell isolates; **P<0.01; ***P<0.001).

Individual knockdown of RB1, p107 or p130 in cultured human fibroblasts results in p16 induction.^15^ In contrast, we demonstrate that suppression of p16 expression in the lung epithelium *in vivo* is unique to RB1. p16 protein and messenger RNA levels were increased in RB1-deficient lungs in a conditional mouse model wherein *Rb1* ablation was targeted to the lung epithelium, but were not induced in p107^−/−^ or p130^−/−^ lungs (Figures 1a–c).^11, 12^ Increased p16 expression occurred by 4–5 weeks of age with elevated p16 protein levels being maintained in 8–9-month-old lungs (Figure 1b). Previous studies localized p16 protein expression to conducting airway Club cells and distal alveolar epithelial type II cells; two progenitor cell types believed to be cells of origin for NSCLC.^11, 13, 14^ p16 message was induced ninefold in RB1-deficient primary type II cell isolates as compared to RB1-proficient control cells consistent with RB1 mediating suppression of p16 at the transcriptional level (Figure 1c). In contrast, induction of p16 messenger RNA was not seen in p107^−/−^ or p130^−/−^ primary type II cells (Figure 1c). Together, these data demonstrate that p16 repression is a unique RB1 function in the lung epithelium and identify a fundamental difference between p16/RB1 pathway regulation in lung epithelial cells *in vivo* and fibroblasts in culture. Given that RB1, but not p107 or p130, is a *bona fide* tumor suppressor, we tested the hypothesis that the unique p16 upregulation after RB1 loss functioned to regulate epithelial cell growth and protect against carcinogenesis.

### p16 induction after RB1 loss does not lead to cellular senescence but rather is critical for promoting epithelial cell growth

Similar to activated oncogenes, loss of tumor suppressor expression activates senescence to limit tumorigenesis *in vivo*.^23^ Senescent cells rely heavily on persistent p16 expression to maintain the senescent state.^18^ RB1 loss in the thyroid results in cellular senescence with p16 induction being important for antagonizing thyroid carcinogenesis.^19^ However, in contrast to the thyroid, cellular senescence was not induced in RB1-deficient lung epithelial cells despite robust p16 expression. Senescence-associated β-galactosidase activity, the most widely accepted biomarker of cellular senescence, was not detected in the RB1-deficient lung epithelium (Figure 1d) differing from the detection of this biomarker in the RB1-deficient thyroid.^18, 19, 23^ Exit from the cell cycle is the central characteristic of cellular senescence defined as a stress-induced irreversible proliferative arrest with resistance to mitogenic and oncogenic stimuli.^18, 23^ Consistent with p16 induction not being associated with cellular senescence, RB1-deficient lung epithelial cells proliferated in primary cultures and after cytotoxic injury *in vivo* to regenerate the pulmonary epithelium (Figure 1e).^24^ Taken together, these data indicate that p16 induction is not sufficient to induce cellular senescence in the RB1-deficient lung epithelium, and unlike in the thyroid is not associated with a cellular senescence state.

To directly assess the biologic significance of p16 induction in RB1-deficient epithelial cells, we generated mice with RB1-deficient lung epithelium in a p16^−/−^ background and compared the growth of combined RB1/p16-deficient primary epithelial type II progenitor cells to control RB1/p16-proficient cells as well as cells lacking RB1 or p16 alone. RB1 loss resulted in increased epithelial cell growth as compared to RB1 and p16-proficient control cells (Figure 1e). This is in direct contrast to murine and human fibroblasts wherein RB1 family proteins have redundant functions with loss of all three pocket proteins being required to increase fibroblast growth.^20, 21, 22^

Based on the well-established growth suppressive functions of p16, it was expected that p16 loss would enhance RB1-deficient cell growth. Surprisingly, loss of p16 suppressed growth of RB1-deficient epithelial cells demonstrating that p16 was required for growth of RB1-deficient lung epithelial cells. Cells from littermate p16^−/−^ lungs lacking one or both transgenes required for *Rb1* ablation had growth rates similar to control cells indicating that p16 loss alone did not alter lung epithelial cell growth. Taken together these data identify a novel growth-enhancing function for p16 in RB1-deficient cells. Moreover, the data demonstrate fundamental differences in p16/RB1 pathway mediated control of epithelial versus fibroblast cell growth highlighting the importance of defining p16 functions in epithelial cells that give rise to cancer.

### p16 enhances growth of RB1-deficient cells by enhancing cell survival

**Figure 2:**
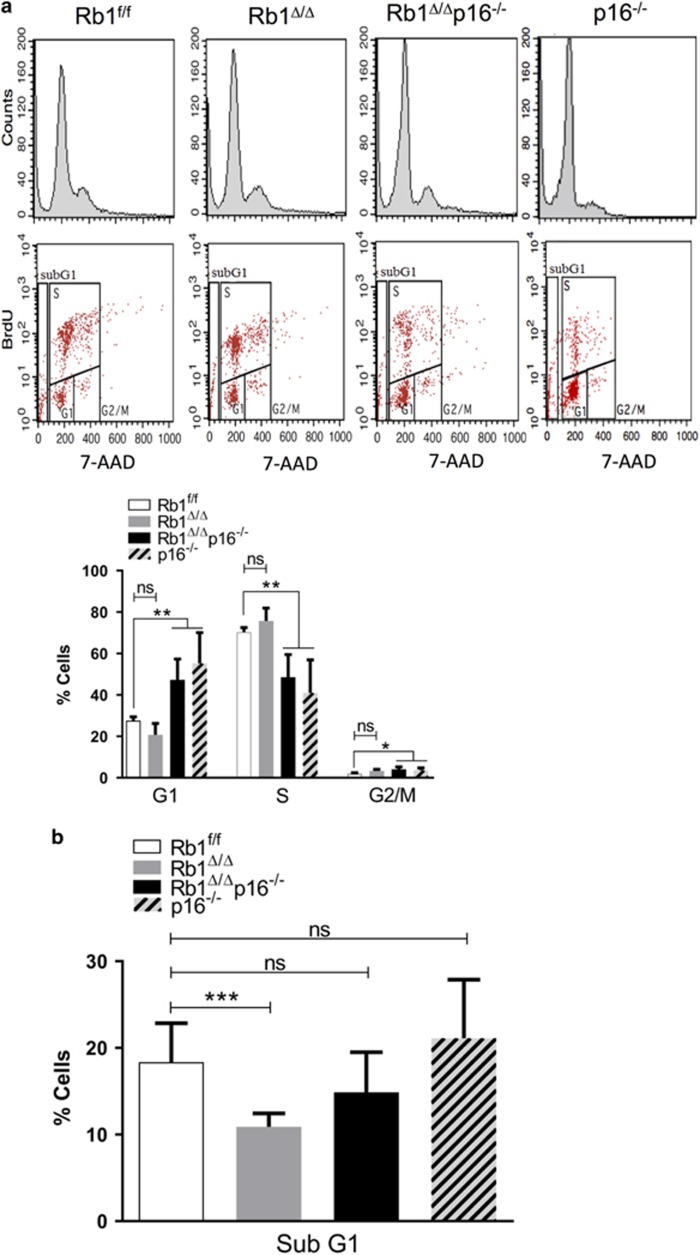
p16 enhances cell cycle progression and survival of RB1-deficient type II cells. Representative cell cycle profiles (top) and quantifications (bottom) of flow cytometric analyses of Rb1^f/f^, Rb1^Δ/Δ^, Rb1^Δ/Δ^p16^−/−^ and p16^−/−^ primary type II cells harvested after 120 h in culture, BrdU labeling for 18 h and 7-AAD staining. (**a**) RB1 loss did not result in statistically significant changes in cell cycle progression as compared to control RB1. p16 loss, irrespective of Rb1 status, was associated with cell cycle profile alterations including decreased S-phase and increased G1 and G2/M phase cells without a change in overall cell growth as shown in Figure 1e (mean±s.d.; *n*=5–10 wells from 3 to 4 independent cell isolates; *P<0.05, **P<0.01). (**b**) Rb1 loss led to a significant reduction of subG1 cells as compared to Rb^f/f^ control cells with additional loss of p16 restoring the percentage of subG1 cells to Rb1/p16-proficient control levels. p16 loss alone did not alter the fraction of subG1 cells as compared to Rb^f/f^ control cells (mean±s.d.; *n*=9–18 wells from 4 to 5 independent cell isolates; ***P<0.001).

To identify the mechanism underlying p16-dependent promotion of RB1-deficient epithelial cell growth, cell cycle analysis was performed on primary lung epithelial type II cells of the defined genotypes. RB1 loss was associated with a slight increase in proliferating S-phase cells that did not reach statistical significance (Figure 2a) indicating that enhanced proliferation alone did not account for the increased growth of RB1-deficient epithelial cells (Figure 1e). Instead, enhanced RB1-deficient cell growth was due to increased cell survival that was dependent upon p16 (Figure 2b). Accordingly, additional loss of p16 led to increased death of RB1-deficient cells back to control levels (Figure 2b). p16 loss alone has no effect on lung epithelial cell survival but resulted in an altered cell cycle profile with a decreased proportion of cells in S-phase and more cells in G1 and G2/M (Figures 2a and b). The cell cycle profile alterations in p16^−/−^ cells were independent of RB1 status and likely reflect changes in cell cycle kinetics given that p16 loss alone did not alter lung epithelial cell growth as assessed by cell number (Figure 1e). Taken together, the data demonstrate that RB1 and p16 loss have distinct effects on lung epithelial cell growth and that p16 functions to enhance growth of RB1-deficient lung epithelial cells. Moreover, the studies identify enhanced cellular survival as the mechanism by which RB1 loss and p16 promote lung epithelial cell growth.

### p16 suppresses immortalization of RB1-deficient cells

**Figure 3:**
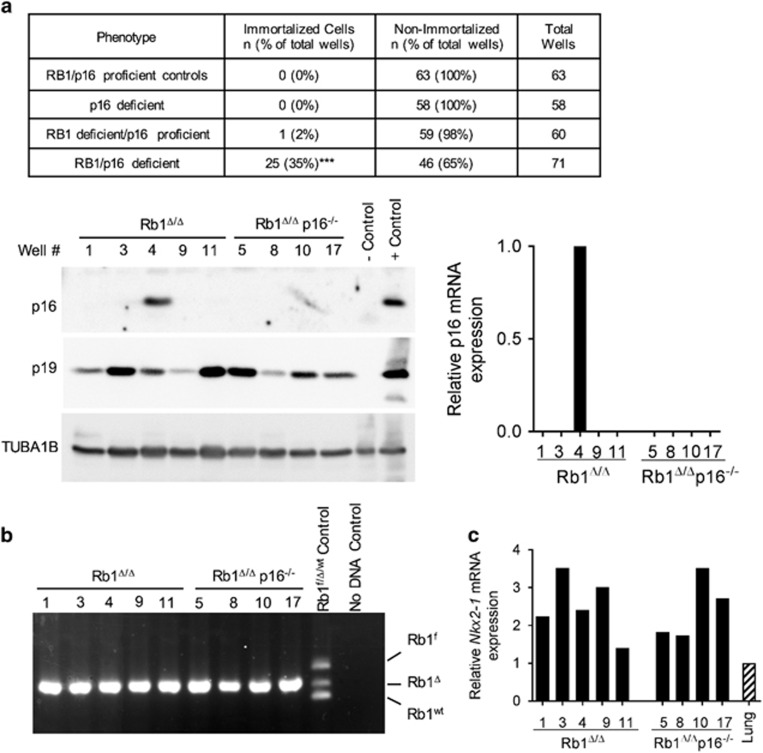
p16 suppresses immortalization of RB1-deficient cells. (**a**) Summary of percentage of tissue culture plate wells seeded with Rb1^f/f^, Rb1^Δ/Δ^, Rb1^f/f^p16^−/−^ and Rb1^Δ/Δ^p16^−/−^ primary type II cells that resulted in immortalized cell populations (data are representative of three independent cell isolates and experiments; ***P<0.001 by Fisher’s exact test for RB1/p16 deficient vs all other groups). Representative western blot (left) and quantitative reverse transcription PCR (RT-PCR; right) analyses demonstrate that only 1 out of 5 immortalized cell populations derived from Rb1^Δ/Δ^ type II cell cultures retained p16 expression and are thus included as RB1/p16 deficient in the summary. p19 was expressed in all cell populations. Lysates were evenly loaded as assessed by reprobing for TUBA1B. p16^−/−^ lung and p16-positive mouse tumor tissue served as negative and positive controls, respectively. Well # represents distinct cell populations derived from individual wells. (**b**) Representative PCR analysis on DNA from Rb1^Δ/Δ^p16^−/−^ immortalized cells demonstrating all immortalized cells populations had Rb1 recombination (Rb1^Δ^) without detection of floxed (Rb1^f^) or wild-type (Rb1^wt^) alleles confirming derivation from Rb1 ablated lung epithelium. (**c**) All immortalized type II cell populations expressed the lung epithelial cell marker, Nkx2-1, at similar or higher levels than lung tissue as assessed by quantitative real-time RT-PCR.

Primary epithelial type II progenitor cells that serve as cells of origin for lung cancer were isolated from RB1/p16 deficient, p16 deficient, RB1 deficient and control RB1/p16-proficient lungs and serially passaged in culture using a modified 3T3 protocol to determine the role of RB1 and p16 in suppressing epithelial immortalization. Despite decreased growth of combined RB1/p16-deficient cells in short-term primary cultures, p16 loss resulted in increased immortalization of RB1-deficient lung epithelial progenitor cells in long-term culture. Twenty-five immortalized cell populations were established from RB1/p16-deficient cells, whereas only one immortalized RB1-deficient cell population that retained p16 expression was established, and no immortalized cell populations were obtained from p16^−/−^ or control RB1/p16-proficient cells (Figure 3a). Primary type II cells of all genotypes underwent a progressive decline in growth within the first 2–3 successive transfers with the first sign of emergence as established lines being an increase in growth rate resulting in cell numbers exceeding that in the original culture. The growth rate has remained constant or continued to increase in the 10 cell populations that have been propagated for >10 passages to date, including two established cell lines propagated for >60 passages. Recombination of the floxed *Rb1* allele and expression of the lung epithelial cell marker, *Nkx2-1*, was detected in all established cell populations confirming the lung epithelial cell lineage (Figures 3b and c). Interestingly, 80% (4/5) of the immortalized cell populations established from Rb1-deficient lungs lacked p16 messenger RNA and protein expression (Figure 3a) providing further evidence that p16 is critical for suppressing immortalization of RB1-deficient epithelial cells.

TP53 mutations are commonly detected in human cancers, including lung cancer, and spontaneous cellular immortalization is frequently associated with loss of TP53 and/or p19^Arf^.^25^ In contrast to other cell types, immortalization of RB1/p16-deficient cells was not associated with loss of Trp53 or p19^Arf^ (Figure 3a and Supplementary Figure). Activated Trp53 as assessed by serine 15 phosphorylation was detected in 23 independently derived immortalized cell populations demonstrating maintained RB1/p16-deficient lung epithelial progenitor cell growth even in the presence of Trp53 activation (Supplementary Figure). Trp53 exome sequencing of 23 distinct cell populations detected a single missense G1187C mutation in exon 10 in one cell population resulting in the amino acid change, A344P (A347P in human; see Supplementary Figure and Supplementary Table). This deleterious missense mutation inhibits tetramer formation and lacks transcriptional activity but is infrequently mutated in human tumors being reported in only a rare liver cancer in the International Agency for Research on Cancer TP53 database.^26, 27^ Taken together, these data demonstrate that p16 suppresses immortalization of RB1-deficient lung epithelial progenitor cells, and that epithelial immortalization after p16 loss does not require loss of p19^Arf^ or Trp53.

### p16 protects RB1-deficient cells from DNA damage

**Figure 4:**
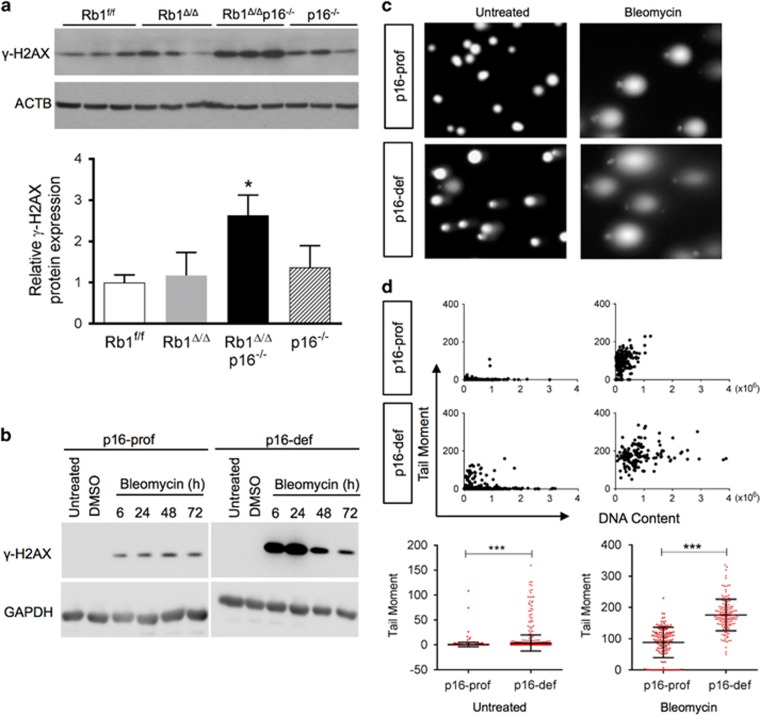
p16 protects RB1-deficient cells from DNA damage. (**a**) Expression of the DNA damage marker, γ-H2AX, was increased in Rb1^Δ/Δ^p16^−/−^ type II cell isolates as compared to Rb1^f/f^, Rb1^Δ/Δ^ and p16^−/−^ cells by western blot analysis with quantification by relative densitometric values normalized to ACTB. Lysates were evenly loaded as assessed by reprobing for ACTB (mean±s.d.; *n*=3 isolates from two independent experiments per group; *P<0.05). (**b**) γ-H2AX induction after bleomycin treatment was higher in p16 deficient (p16-def) as compared to p16-proficient (p16-pro) cells by western blot analysis of immortalized lung epithelial cells treated with bleomycin for 6, 24, 48 and 72 h. Untreated and DMSO vehicle-treated controls at the 6 h time point are shown. Cell lysates were evenly loaded as assessed by reprobing for GAPDH (representative of three independent experiments). (**c**) Representative comet assay SYBR Green stained images of p16-prof and p16-def cells untreated or treated with 10 μU bleomycin for 24 h. (**d**) Quantification of DNA damage by comet assay tail moments. Scatter plots (top) with quantification (bottom) of untreated and bleomycin-treated p16-prof and p16-def cells (mean±s.d.; *n*=920 total untreated cells and 140–150 representative treated cells per group from three independent experiments; ***P<0.001).

A unifying mechanism whereby p16 could enhance cell growth and protect from immortalization is by maintaining DNA integrity. Accumulation of DNA damage after p16 loss would lead to genetic instability resulting in cellular growth arrest or apoptosis if unrepaired while also predisposing to immortalization by increasing risk for acquiring additional oncogenic mutations. Indeed, p16 loss led to increased DNA damage in primary and immortalized RB1-deficient lung epithelial progenitor cells. Expression of the robust DNA damage marker, γ-H2AX, was significantly increased in combined RB1/p16-deficient primary lung epithelial type II cell isolates (Figure 4a).^28^ In addition, p16-proficient lung epithelial cells incurred less DNA damage than p16-deficient cells when grown in culture as well as after treatment with the DNA damaging chemotherapeutic drug, bleomycin, as assessed by γ-H2AX expression and comet assay (Figures 4b–d).

**Figure 5:**
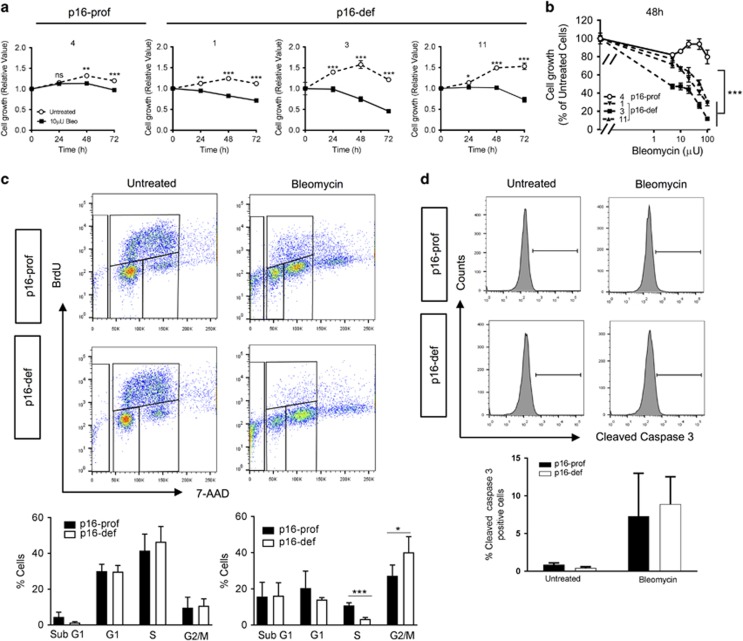
p16 protects RB1-deficient cells from bleomycin-induced cell cycle arrest with increased proliferation. Effects of chemotherapy-induced DNA damage on cell growth was assessed in p16-prof (4) and three p16-def (1, 3, and 11) cell populations. (**a**) Cell growth as assessed by WST1 assay was significantly suppressed after 24–48 h of bleomycin (10 μU) treatment as compared with untreated controls with p16-def cells having more dramatic growth suppression as compared to p16-prof cells (mean±s.d.; *n*=3 wells per group; *P<0.05, **P<0.01, ***P<0.001). (**b**) Bleomycin-induced growth suppression of p16-def cells was dose-dependent with the growth of p16-def cells significantly suppressed at lower bleomycin doses as compared to p16-prof cells (mean±s.e.m.; *n*=3 wells per group; *** *P*<0.001). (**c**) Representative cell cycle profiles (top) and quantifications (bottom) of flow cytometric analyses of untreated and bleomycin-treated (10 μU for 48 h) p16-prof and p16-def cells after BrdU labeling for 3 h and 7-AAD staining. Cell cycle progression was similar in untreated p16-prof and p16-def cells, whereas p16-def cells had a significantly lower percentage of proliferating S-phase cells and increased G2M-phase cells as compared to p16-prof cells after bleomycin treatment (mean±s.d.; *P<0.05, ***P<0.001). (**d**) Representative histograms (top) and quantification (bottom) of cleaved caspase 3-positive cells by flow cytometric analyses showing similar numbers of apoptotic p16-def and p16-prof cells in the absence and presence of bleomycin treatment (mean±s.d.). All results are representative of three independent experiments.

Protection from DNA damage is expected to enhance cell growth and suppress cellular transformation. Indeed, p16-proficient cells had markedly reduced sensitivity to bleomycin-induced growth arrest as compared to p16-deficient cells (Figures 5a–d). Bleomycin treatment inhibited growth of p16-deficient cell populations in a dose-dependent manner, whereas p16-proficient cells had markedly less growth suppression at the same bleomycin doses, indicating reduced sensitivity to chemotherapy-induced DNA damage. Enhanced growth of p16-proficient cells resulted from increased cell cycle progression as indicated by p16 loss leading to decreased S-phase and increased G2/M phase cells without significant alterations in apoptotic cell death (Figures 5c and d). Thus, p16 promotes growth of RB1-deficient lung epithelial progenitor cells by protecting against DNA damage resulting in enhanced cellular proliferation and growth.

### p16 suppresses transformation of RB1-deficient cells

**Figure 6:**
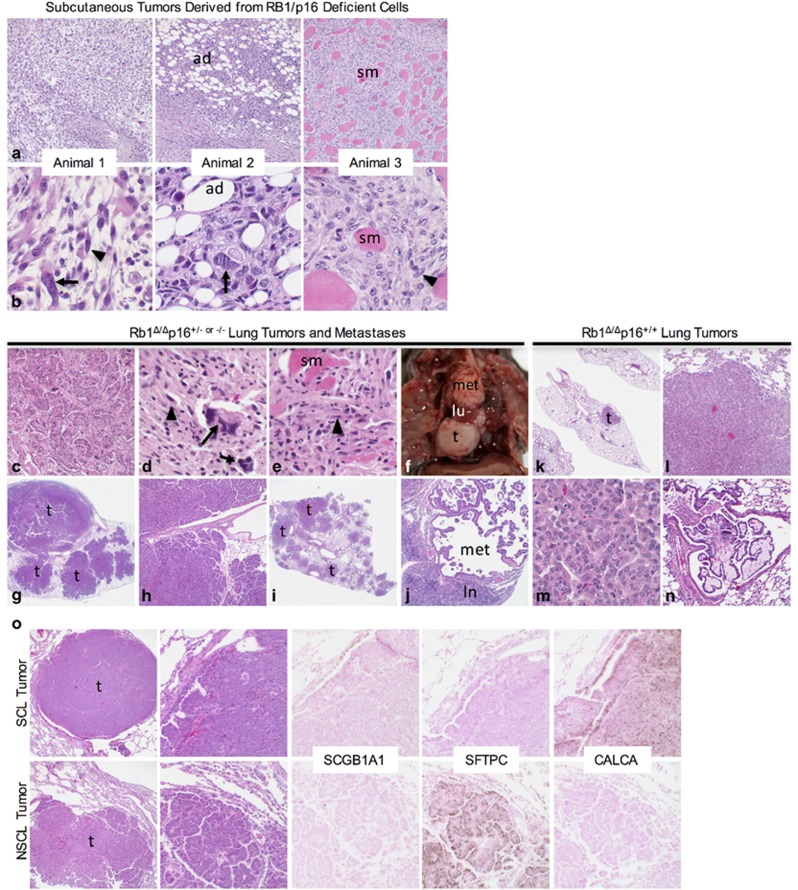
p16 loss promotes transformation and tumorigenesis of RB1-deficient lung epithelial cells. (**a**, **b**) Representative low (**a**) and high (**b**) power images of subcutaneous tumors arising after injection of Rb1/p16-deficient lung epithelial cells into three independent mice showing histologic features of pleomorphic non-small cell lung cancer (NSCLC) including malignant giant cells with markedly enlarged nuclei (arrows) and spindled cells (arrowheads) invading host adipose tissue (ad) and skeletal muscle (sm). (**c**–**j)** Representative lung tumors and metastases in Rb1^Δ/Δ^p16^+/−^ and Rb1^Δ/Δ^p16^−/−^ mice. Pleomorphic NSCLC (**c**) with metastasis to chest wall (**d**, **e**) and histologic features similar to subcutaneous tumors, including giant cells with enlarged nuclei (arrows) and spindled cells (arrowheads) invading skeletal muscle (sm). Gross image of small cell lung cancer (SCLC) (**f**, **t**) replacing right lower lung lobe with adjacent normal middle lung lobe (lu) and mediastinal metastasis (met). Multifocal non-small cell lung (NSCL) tumors with extensive airway invasion (**g**–**h**, t) and a second mouse with multifocal NSCL tumors (**i**, t) including adenocarcinoma metastasis (**j**, met) to mediastinal lymph node (**j**, ln). (**k**–**n**) Representative lung tumors in Rb1^Δ/Δ^p16^+/+^ mice. One or two tumors (**k**, t) per mouse were present with papillary and/or solid growth (**l**) lacking the marked cytological atypia seen in Rb1/p16-deficient tumors (**m**) and only focal invasion of airway (**n**). (**o**) Small cell lung (SCL) and NSCL tumors (t) in a single Rb1^Δ/Δ^p16^+/−^ mouse showing characteristic morphology and immunostaining for CALCA but not SCGB1A1 and SFTPC in SCL tumor and SFTPC but not SCGB1A1 and CALCA in NSCL tumor (representative of 4 SCL and 16 NSCL tumors including 4 metastatic tumors). Original magnifications: × 2 (**g**, **i**, **k**); × 10 (**h** and **o** left column); × 20 (**a**, **c**, **j**, **l**, **n**); × 40 (O right 4 columns); × 100x (**b**, **d**, **e**, **m**).

To test whether increased DNA damage in p16-deficient cells predisposes to cellular transformation, p16-deficient and -proficient RB1 ablated lung epithelial progenitor cells were subcutaneously injected into the flanks of nude mice and assessed for tumor growth. Tumors developed in 22% (7/32) of sites injected with p16-deficient cells whereas no tumors developed at sites injected with cells that retained p16 expression. Tumors developed in four distinct mice representing two independently derived p16-deficient cell populations, and arose after 9–28 week latency periods consistent with the tumors arising as a result of randomly acquired oncogenic mutations. The tumors were comprised of highly pleomorphic epithelioid, spindled and giant cells with aggressive histologic features including invasion of surrounding adipose tissue and skeletal muscle with focal tumor necrosis (Figures 6a and b). The p16-deficient tumors were morphologically similar to human sarcomatoid lung carcinomas, a poorly differentiated NSCLC subtype associated with a worse prognosis.^29^

### p16 suppresses tumorigenesis in RB1-deficient lung epithelia

The role of p16 in suppressing spontaneous tumor formation in RB1-deficient lungs was investigated *in vivo* by generating double transgenic Scgb1a1-rtTA/tetCre mice wherein RB1 ablation was targeted to the lung epithelium in a p16^+/−^ and p16^−/−^ background (Figure 1a). The *Scgb1a1* promoter was chosen for these studies to avoid the high incidence of bilateral medullary thyroid carcinomas that develop in mice with *Sftpc* driven RB1 ablation due to the targeting of a subset of thyroid cells.^30^ The incidence of spontaneous lung tumors was nearly doubled in p16-deficient mice with tumors having a more aggressive phenotype as indicated by increased cytologic atypia as well as invasive growth and metastases which were not seen in p16-proficient mice (Table 1 and Figures 6c–j). Multifocal lung tumors of multiple histologic subtypes developed in p16-deficient mice including SCLC which were never seen or reported in mice with RB1 or p16 loss alone or in RB1/p16-proficient controls (Table 1).^11, 24, 31, 32, 33^ Aggressive sarcomatoid carcinoma with chest wall invasion and metastatic nodules involving the diaphragm developed in a p16-deficient mouse that was morphologically indistinguishable from human sarcomatoid NSCLC and the subcutaneous tumors that developed from RB1/p16-deficient cells (Figures 6a–e). The incidence of multifocal tumors was increased in p16-deficient mice with two mice developing extensive multifocal adenomas and adenocarcinomas that nearly completely replaced the lung lobes with one developing metastasis to a mediastinal lymph node (Table 1 and Figures 6g–j). In contrast, tumors that developed in p16-proficient mice consisted of one or two localized tumors that lacked significant cytologic atypia and did not metastasize (Figures 6k–n). Small cell tumors only developed in combined RB1/p16-deficient lungs (Table 1). Two mice developed metastatic SCLC with mediastinal, chest wall, diaphragm and/or thoracic soft tissue metastases (Figure 6f). Small cell tumors were positive for the neuroendocrine marker, calcitonin related peptide alpha/calcitonin gene-related peptide (CALCA/CGRP), and negative for non-neuroendocrine type II and Club cell markers, SFTPC and SCGB1A1, respectively (Figure 6o). In contrast, the non-small cell lung tumors were positive for SFTPC and negative for CALCA and SCGB1A1, indicating a type II cell phenotype (Figure 6o). Lung tumors were present in mice analyzed at 17–21 months of age indicating a relatively long latency period consistent with p16 loss leading to sporadic accumulation of somatic oncogenic mutations as a result of increased susceptibility to DNA damage. Taken together, these data demonstrate that p16 induction after RB1 loss protects against DNA damage, cellular immortalization and transformation as well as development of aggressive lung tumors.

## Discussion

Loss of RB1 function is considered requisite for cancer development leading to intensive study of the roles and regulation of RB1 in controlling cell cycle progression.^34^ The RB1 family proteins, RB1, p107, and p130, have overlapping and compensatory functions in cell-cycle control. Like RB1, both p107 and p130 associate with E2F transcription factors to transcriptionally suppress genes required for cell cycle progression, interact with tumor virus oncoproteins, and induce cell-cycle arrest when overexpressed.^35^ Yet, despite the structural and functional similarities among the RB1 family proteins, somatic mutations in human cancers are almost exclusively found in *Rb1* providing evidence that RB1 has unique functions in epithelial cells critical for suppressing carcinogenesis.^34^ Importantly, our studies demonstrate that RB1 loss alone is sufficient to drive lung epithelial progenitor cell growth which is in marked contrast to fibroblasts wherein loss of all three RB1 family proteins is required to promote fibroblast growth.^20, 21, 22^ Suppression of RB1, but not p107 or p130, also extends the proliferative lifespan of primary mammary epithelial cells providing further evidence that RB1 has unique growth suppressive functions in epithelial cells that are not shared in fibroblasts.^36^ We also show that p16 suppression is a non-redundant RB1 function in lung epithelial cells *in vivo* that again is distinct from fibroblasts in culture wherein all three RB1 family proteins suppress p16 expression.^15^ Thus, these studies identify critical differences in RB1 family protein function and p16 regulation in epithelial cells and fibroblasts, highlighting the importance of defining p16/RB1 tumor suppressive functions in epithelial cells that give rise to 80% of human adult malignancies.

The current linear paradigm that p16 functions by activating RB1 would predict that p16 induction in RB1-deficient cells is biologically inconsequential. Our results, however, demonstrate that p16 is biologically active in RB1-deficient lung epithelial cells. Notably, induction of p16 in RB1-deficient lung epithelium did not induce senescence but rather promoted cell survival and protected against DNA damage. Our data in combination with reports in the literature clearly demonstrate that p16 function in RB1-deficient epithelial cells is dependent upon cellular context. Similar to lung epithelial cells, p16 is critical for survival of RB1-deficient human papilloma virus-positive cervical cancer cell lines.^37^ However in contrast to the lung, p16 induction in the RB1-deficient thyroid is associated with cellular senescence and decreased epithelial cell growth. Interestingly, p16 protects against genetic instability to enhance survival and proliferation of hematopoietic progenitor cells demonstrating that this p16 function is not restricted to the lung.^38^ Loss of the telomere DNA-binding protein, protection of telomere 1 (POT1), results in DNA damage leading to fatal bone marrow failure.^38^ Induction of p16 in hematopoietic progenitor and stem cells in POT1 deficient mice protected against dysfunctional telomere induced DNA damage resulting in increased lifespan due to increased hematopoietic progenitor cell survival and proliferative capacity. Consequentially, loss of p16 resulted in genetic instability and ectopic apoptotic cell death similar to that seen in the current studies. Thus, unlike in the thyroid and in aging tissues wherein p16 accumulation results in cellular senescence, p16 accumulation in the setting of oncogenic events like RB1 loss, human papilloma virus infection and telomere dysfunction has important cytoprotective functions.^39, 40, 41, 42^ Together, these findings highlight the importance of taking into account the cellular context when interpreting physiologic functions of p16. In addition, the data directly demonstrate that p16 is biologically active in RB1-deficient lung epithelial cells, functioning to protect against DNA damage and cell death through mechanisms that do not require RB1.

p16 positively regulates all three Rb family proteins raising the possibility that p16 effects in RB1-deficient cells are mediated through p107 and/or p130. The phenotypes of RB1/p16 lungs in the current studies, however, differ markedly from phenotypes in combined RB1 family deficient lungs demonstrating that p16 effects cannot be explained by loss of RB1 family function alone. Ablation of all three RB1 family proteins in the lung epithelium leads to development of neuroendocrine tumorlets that do not progress to SCLC and no NSCL tumors.^43^ Thus, additional oncogenic events are required for development of the SCLC and NSCL tumors present in RB1/p16-deficient lungs. Combined RB1/p130 ablation using the same murine model as in the current studies was not sufficient to induce lung tumors.^11^ In addition, combined RB1/p107 loss led to an increased incidence of NSCL tumors that lacked the aggressive metastatic phenotype of RB1/p16-deficient tumors and was not sufficient to induce SCLC.^11^ Taken together these studies indicate that p16 effects in RB1-deficient cells cannot be solely explained by loss of RB1 family function. Thus, the current genetically engineered mice represent a tractable model to identify these critical p16 tumor suppressive functions that are not mediated through RB1.

While loss-of-function *RB1* mutations are universally detected in retinoblastoma and SCLC, RB1 is much more frequently inactivated in human tumors by p16 loss providing evidence that p16 also has critical tumor suppressive functions that are not mediated through RB1 in humans. In the lung, the p16 locus is inactivated in 43% of adenocarcinomas and 72% of squamous cell carcinomas that comprise the vast majority of NSCLC.^7, 8^ Molecularly targeted therapies have dramatically improved treatment for NSCLC patients whose tumors have somatically activated oncogenes such as epidermal growth factor receptor (EGFR), translocated anaplastic lymphoma kinase (ALK), and serine/threonine-protein kinase B-Raf (BRAF).^13, 44^ However, there are currently no targeted therapies for the frequent tumors with p16 inactivation highlighting the need to define critical p16 tumor suppressive functions in the lung epithelium to guide rationale development of new therapeutic strategies. We demonstrate that p16 functions to protect against DNA damage in lung epithelial progenitor cells that give rise to NSCLC. Genetic instability, as was seen in lung epithelial cells after p16 loss, is a defining characteristic of cancer that promotes tumor initiation and disease progression.^45^ These findings are of direct relevance to human lung cancers wherein p16 loss occurs as an early event in carcinogenesis and is positively associated with dose and duration of exposure to tobacco smoke, a potent mutagen.^46^ Over 80% of lung cancers are associated with smoking and thus occur in a mutagenic environment wherein susceptibility to genetic instability would significantly increase cancer risk. SCLC and NSCLC are both associated with smoking and developed after p16 loss providing evidence that p16 functions to suppress tumorigenesis through RB1 independent mechanisms in epithelial cell lineages that give rise to both neuroendocrine and non-neuroendocrine cancers.^13, 47, 48^ Although p16 loss is not detected in human SCLC, these studies indicate that mechanisms underlying p16-dependent protection against DNA damage and carcinogenesis are shared in epithelial cells that give rise to both NSCLC and SCLC. Thus the molecular mechanisms underlying p16 protection of genetic stability may have general relevance to lung carcinogenesis and response to DNA damaging therapeutics.

In summary, the current studies directly demonstrate that p16 suppresses carcinogenesis through mechanisms that do not require RB1 function. Induction of p16 in RB1-deficient lung epithelial cells is not associated with cellular senescence as reported in other tumors and with cellular aging, but rather is critical for maintaining genomic integrity. Consequently, p16 loss results in epithelial cell death while also predisposing to cellular immortalization and transformation after a long latency, consistent with p16 protecting against DNA damage and subsequent accumulation of oncogenic mutations. The RB1 independent tumor suppressive functions of p16 are physiologically relevant *in vivo* as demonstrated by the development of aggressive lung cancers upon p16 loss in RB1-deficient lungs of genetically engineered mouse models. Together, these studies identify p16 loss as a molecular event that causes genetic instability in addition to inactivating RB1 function thus providing an explanation for why p16 is preferentially targeted in human cancers.

## Materials and methods

### Mouse generation

Sftpc-rtTA/tetCre Rb1^f/f^, Scgb1a1-rtTA/tetCre Rb1^f/f^ and p16^−/−^ mice were mated to generate the indicated genotypes. RB1 ablation was induced by doxycycline treatment and genotypes confirmed as previously described.^11, 12, 24, 32^

### Primary type II cell isolation, immortalization, transformation and growth assays

Primary type II cell cultures were isolated from lungs of 5–6-week-old mice, plated on transwell clear polyester membrane inserts covered with Cultrex basement membrane (Trevigen, Gaithersburg, MD, USA) in 6-well plates (Corning, Corning, NY, USA) and grown in Bronchial Epithelium Growth Medium (BulletKit, Lonza, Walkersville, MD, USA) supplemented with Penicillin/Streptomycin 50 × solution (1:100).^49^ Media was changed each day for the cell growth and cell cycle profile studies. Cells were removed with Dispase (BD Pharmingen, San Jose, CA, USA). Immortalization was assessed using a modified 3T3 protocol plating 3.5 × 10^3^ cells per well in 12-well plates and passaging every 3 weeks with trypsin.^50^ The entire cell population was plated in subsequent passages if total cell count was ⩽3.5 × 10^3^ cells and media was changed without trypsinization in wells with ⩽20 cells on visual inspection. Cells were grown in RPMI-1640 media supplemented with 10 nM Hydrocortisone, 0.005 mg/ml Insulin, 0.01 mg/ml Transferrin, 10 nM Beta-estradiol, 30 nM Sodium selenite (HITES), 10 nM HEPES, 2 nM L-glutamine, 100 U/ml Penicillin, 0.1 mg/ml Streptomycin and 5% FBS for 33 weeks. Media was changed every week or more frequently if needed. Cells from an individual well were considered independently derived immortalized cell populations when cells emerged with constant or rising growth after a marked initial decline in growth that in all cases except one resulted in total cell numbers below the initial 3.5 × 10^3^ cells plated. Immortalized cell populations could also be preserved by freezing with continued propagation after reestablishment in culture. Cellular transformation was assessed by subcutaneous injection of 4.1–5.3 × 10^6^ cells into the flanks of <15 week-old female athymic NCr-nu/nu mice (Comprehensive Mouse and Cancer Core at Cincinnati Children’s Hospital Medical Center) and monitoring for tumor formation. Cell growth was determined by WST1 assays (Millipore, Bedford, MA, USA). Bleomycin treatments consisted of seeding 96-well plates (5 × 10^3^ cells/well) and treating with bleomycin the following day. Untreated or dimethyl sulfoxide vehicle-treated cells served as controls.

### Western blot analysis

Western blot analysis was performed as described^11^ with nitrocellulose membranes probed for p16 (m156, 1:1000, Santa Cruz Biotechnology, Santa Cruz, CA, USA), phosphorylated p53 (9284, 1:2000, Cell Signaling, Danvers, MA, USA), total p53 (CM5, 1:2000, Leika, Biosystems, Richmond, IL, USA), phospho-histone γ-H2AX (1:1000, Upstate, Cell Signaling Solutions, Temecula, CA, USA), p19^ARF^ (sc-32748, 1:500, Santa Cruz Biotechnology), TUBA1B (DM 1A, 1:2500, Sigma-Aldrich Chemical Company, St Louis, MO, USA), GAPDH (8884, 1:10000, Cell Signaling) and/or ACTB (A5060, 1:1000, Sigma-Aldrich Chemical Company).

### Histology and immunohistochemistry

Tissues were analyzed using established protocols^11^ and antibodies CALCA (1:10000; Sigma-Aldrich Chemical Company), SFTPC (pro-SPC; 1:3000, Seven Hill; Cincinnati, OH, USA), SCGB1A1 (1:2000, Santa Cruz, Biotechnology).

### Quantitative real-time reverse transcription PCR

RNA was isolated using Qiagen RNeasy plus Mini Kit. Real-time reverse transcription PCR was performed and analyzed as described.^11^ Taqman primers included assay probe set Nkx2-1, Mm004923451_m1, and p16 specific custom set Forward-CAACGCCCCGAACTCTTTC, Reverse-AAGAGCTGCTACGTGAACGT and Probe-CCCGATTCAGGTGATGAT.

### Senescence-associated ß-galactosidase staining

Lungs were infused with a 1:1 mix of OCT compound and 30% sucrose/PBS/2 mm MgCl_2_. Lung lobes were bisected, immersed in OCT and flash frozen in cryomolds. Frozen 5 μm sections were fixed in 0.2% paraformaldehye in PBS for 10 min, washed thrice and immersed into SA-ß-gal staining solution for 18 h at 37 °C. Slides were washed with PBS/2 mm MgCl_2_ and counterstained with nuclear fast red. SA-ß-gal staining solutions: 40 mm citric acid/Na_2_HPO_4_ buffer (adjusted accordingly for pH 6.0 or pH 4.0), 150 mm NaCl, 2 mm MgCl_2_, 5 mm Potassium Ferricyanide, 5 mm Potassium Ferrocyanide and 1 mg/ml X-gal.

### Flow cytometry

Cell cycle profiles were assessed by flow cytometry after labeling for 18 h (primary cells) or 3 h (immortalized cells) with 10 μm bromodeoxyuridine (BrdU, BD Pharmingen) and staining with fluorescein isothiocyanate-conjugated anti-BrdU antibody and 7-AAD (BD Pharmingen). Cell death was determined by quantifying subG1 fractions and fluorescein isothiocyanate Cleaved caspase 3-stained cells (BD Pharmingen). Analysis was performed on a BD FacsCanto and data analyzed on FlowJo software.

### Comet assay

Cells were trypsinized, resuspended in 4% FBS in PBS (3 × 10^5^ cells/ml), embedded in liquefied 0.75% low melting point agarose and transferred onto slides. After solidification at 4 °C, cells were placed in lysis (2.5 m NaCl, 100 mm EDTA, 10 mm Tris Base and 1% sodium lauryl sulfate) and alkali (200 mm NaOH and 1 mm EDTA) solutions followed by electrophoresis in alkali solution for 15 min at 1 V/cm. After staining with SyBR Green solution, comet tails were photographed using Olympus IX60 System Microscope (Olympus, Pittsburgh, PA, USA) and Image ProPlus 7 software and measured using CometScore software. Tail moments were defined as percentage DNA in the comet tail multiplied by tail length.

### Statistical analyses

Student *t*-test and one-way ANOVA followed by Tukey’s multiple comparisons or Dunnett’s test was used to analyze statistical significance between two groups or multiple groups, respectively. Fisher’s exact test was used for analyzing genotypic differences in cellular immortalization and transformation. Significance was indicated by *P*<0.05. Statistical tests were performed using GraphPad Prism version 6.07 for Windows and GraphPad Software (La Jolla, CA, USA).

## Figures and Tables

**Table 1:** Lung tumor incidence and phenotypes

	*Rb1 ablated*		
	*p16*^*+/+*^	*p16*^*+/−*^	*p16*^*−/−*^
Total animals	37	36	26
Animals with lung tumors (%)	6 (16)	10 (28)	8 (31)
Multifocal tumors (%)	3 (8)	5 (14)	4 (15)
Total tumors	9	21	17
SCL tumors (%)	0 (0)	2 (10)	4 (24)
NSCL tumors (%)	9 (100)	19 (90)	13 (76)
Metastatic tumors (%)	0 (0)	3 (14)	1 (6)

Abbreviations: NSCL, non-small cell lung; SCL, small cell lung.

## References

[bib1] Ciriello G, Miller ML, Aksoy BA, Senbabaoglu Y, Schultz N, Sander C. Emerging landscape of oncogenic signatures across human cancers. Nat Genet 2013; 45: 1127–1133. 24071851 10.1038/ng.2762 PMC4320046

[bib2] Malumbres M, Barbacid M. To cycle or not to cycle: a critical decision in cancer. Nat Rev Cancer 2001; 1: 222–231. 11902577 10.1038/35106065

[bib3] Knudsen ES, Knudsen KE. Retinoblastoma tumor suppressor: where cancer meets the cell cycle. Exp Biol Med 2006; 231: 1271–1281. 10.1177/153537020623100713 16816134

[bib4] George J, Lim JS, Jang SJ, Cun Y, Ozretic L, Kong G et al. Comprehensive genomic profiles of small cell lung cancer. Nature 2015; 524: 47–53. 26168399 10.1038/nature14664 PMC4861069

[bib5] Zhang J, Benavente CA, McEvoy J, Flores-Otero J, Ding L, Chen X et al. A novel retinoblastoma therapy from genomic and epigenetic analyses. Nature 2012; 481: 329–334. 22237022 10.1038/nature10733 PMC3289956

[bib6] Cooper WA, Lam DCL, O’Toole SA, Minna JD. Molecular biology of lung cancer. J Thorac Dis 2013; 5: S479–S490. 24163741 10.3978/j.issn.2072-1439.2013.08.03 PMC3804875

[bib7] Cancer Genome Atlas Research Network. Comprehensive genomic characterization of squamous cell lung cancers. Nature 2012; 489: 519–525. 22960745 10.1038/nature11404 PMC3466113

[bib8] Cancer Genome Atlas Research Network. Comprehensive molecular profiling of lung adenocarcinoma. Nature 2014; 511: 543–550. 25079552 10.1038/nature13385 PMC4231481

[bib9] Siegel RL, Miller KD, Jemal A. Cancer statistics, 2016. CA Cancer J Clin 2016; 66: 7–30. 26742998 10.3322/caac.21332

[bib10] Meuwissen R, Linn SC, Linnoila RI, Zevenhoven J, Mooi WJ, Berns A. Induction of small cell lung cancer by somatic inactivation of both Trp53 and Rb1 in a conditional mouse model. Cancer Cell 2003; 4: 181–189. 14522252 10.1016/s1535-6108(03)00220-4

[bib11] Simpson DS, Mason-Richie NA. Retinoblastoma family proteins have distinct functions in pulmonary epithelial cells *in vivo* critical for suppressing cell growth and tumorigenesis. Cancer Res 2009; 69: 8733–8741. 19887614 10.1158/0008-5472.CAN-09-1359 PMC2778863

[bib12] Wikenheiser-Brokamp KA. Rb family proteins differentially regulate distinct cell lineages during epithelial development. Development 2004; 131: 4299–4310. 15294860 10.1242/dev.01232

[bib13] Chen Z, Fillmore CM, Hammerman PS, Kim CF, Wong KK. Non-small-cell lung cancers: a heterogeneous set of diseases. Nat Rev Cancer 2014; 14: 535–546. 25056707 10.1038/nrc3775 PMC5712844

[bib14] Sutherland KD, Berns A. Cell of origin of lung cancer. Mol Oncol 2010; 4: 397–403. 20594926 10.1016/j.molonc.2010.05.002 PMC5527931

[bib15] Kotake Y, Cao R, Viatour P, Sage J, Zhang Y, Xiong Y. pRB family proteins are required for H3K27 trimethylation and Polycomb repression complexes binding to and silencing p16INK4alpha tumor suppressor gene. Genes Dev 2007; 21: 49–54. 17210787 10.1101/gad.1499407 PMC1759899

[bib16] Sano T, Oyama T, Kashiwabara K, Fukuda T, Nakajima T. Expression status of p16 protein is associated with human papillomavirus oncogenic potential in cervical and genital lesions. Am J Pathol 1998; 153: 1741–1748. 9846965 10.1016/S0002-9440(10)65689-1 PMC1866324

[bib17] Weinberger PM, Yu Z, Haffty BG, Kowalski D, Harigopal M, Brandsma J et al. Molecular classification identifies a subset of human papillomavirus—associated oropharyngeal cancers with favorable prognosis. J Clin Oncol 2006; 24: 736–747. 16401683 10.1200/JCO.2004.00.3335

[bib18] Sharpless NE, Sherr CJ. Forging a signature of *in vivo* senescence. Nat Rev Cancer 2015; 15: 397–408. 26105537 10.1038/nrc3960

[bib19] Shamma A, Takegami Y, Miki T, Kitajima S, Noda M, Obara T et al. Rb Regulates DNA damage response and cellular senescence through E2F-dependent suppression of N-ras isoprenylation. Cancer Cell 2009; 15: 255–269. 19345325 10.1016/j.ccr.2009.03.001

[bib20] Sage J, Mulligan GJ, Attardi LD, Miller A, Chen S, Williams B et al. Targeted disruption of the three Rb-related genes leads to loss of G(1) control and immortalization. Genes Dev 2000; 14: 3037–3050. 11114892 10.1101/gad.843200 PMC317090

[bib21] Dannenberg JH, van Rossum A, Schuijff L, te Riele H. Ablation of the retinoblastoma gene family deregulates G(1) control causing immortalization and increased cell turnover under growth-restricting conditions. Genes Dev 2000; 14: 3051–3064. 11114893 10.1101/gad.847700 PMC317114

[bib22] Chicas A, Wang X, Zhang C, McCurrach M, Zhao Z, Mert O et al. Dissecting the unique role of the retinoblastoma tumor suppressor during cellular senescence. Cancer Cell 2010; 17: 376–387. 20385362 10.1016/j.ccr.2010.01.023 PMC2889489

[bib23] Kuilman T, Michaloglou C, Mooi WJ, Peeper DS. The essence of senescence. Genes Dev 2010; 24: 2463–2479. 21078816 10.1101/gad.1971610 PMC2975923

[bib24] Mason-Richie NA, Mistry MJ, Gettler CA, Elayyadi A, Wikenheiser-Brokamp KA. Retinoblastoma function is essential for establishing lung epithelial quiescence after injury. Cancer Res 2008; 68: 4068–4076. 18519665 10.1158/0008-5472.CAN-07-5667 PMC2518963

[bib25] Harvey DM, Levine AJ. p53 alteration is a common event in the spontaneous immortalization of primary BALB/c murine embryo fibroblasts. Genes Dev 1991; 5: 2375–2385. 1752433 10.1101/gad.5.12b.2375

[bib26] Bouaoun L, Sonkin D, Ardin M, Hollstein M, Byrnes G, Zavadil J et al. TP53 variations in human cancers: new lessons from the IARC TP53 database and genomics data. Hum Mutat 2016; 37: 865–876. Database version R18, April 2016. 27328919 10.1002/humu.23035

[bib27] Kawaguchi T, Kato S, Otsuka K, Watanabe G, Kumabe T, Tominaga T et al. The relationship among p53 oligomer formation, structure and transcriptional activity using a comprehensive missense mutation library. Oncogene 2005; 24: 6976–6981. 16007150 10.1038/sj.onc.1208839

[bib28] Mah LJ, El-Osta A, Karagiannis TC. gammaH2AX: a sensitive molecular marker of DNA damage and repair. Leukemia 2010; 24: 679–686. 20130602 10.1038/leu.2010.6

[bib29] Stephen B, Edge DRB, Compton CC, Fritz AG, Greene FL, Totti AIII Cancer Staging Handbook From the AJCC Cancer Staging Manual, 7th edn, Springer-Verlag: New York, NY, USA, 2010.

[bib30] Akeno N, Miller AL, Ma X, Wikenheiser-Brokamp KA. p53 suppresses carcinoma progression by inhibiting mTOR pathway activation. Oncogene 2015; 34: 589–599. 24469052 10.1038/onc.2013.589 PMC4112184

[bib31] Sharpless NE, Alson S, Chan S, Silver DP, Castrillon DH, DePinho RA. p16(INK4a) and p53 deficiency cooperate in tumorigenesis. Cancer Res 2002; 62: 2761–2765. 12019151

[bib32] Sharpless NE, Bardeesy N, Lee KH, Carrasco D, Castrillon DH, Aguirre AJ et al. Loss of p16Ink4a with retention of p19Arf predisposes mice to tumorigenesis. Nature 2001; 413: 86–91. 11544531 10.1038/35092592

[bib33] Krimpenfort P, Quon KC, Mooi WJ, Loonstra A, Berns A. Loss of p16Ink4a confers susceptibility to metastatic melanoma in mice. Nature 2001; 413: 83–86. 11544530 10.1038/35092584

[bib34] Burkhart DL, Sage J. Cellular mechanisms of tumour suppression by the retinoblastoma gene. Nat Rev Cancer 2008; 8: 671–682. 18650841 10.1038/nrc2399 PMC6996492

[bib35] Mulligan G, Jacks T. The retinoblastoma gene family: cousins with overlapping interests. Trends Genet 1998; 14: 223–229. 9635405 10.1016/s0168-9525(98)01470-x

[bib36] Bazarov AV, Lee WJ, Bazarov I, Bosire M, Hines WC, Stankovich B et al. The specific role of pRb in p16(INK4A)-mediated arrest of normal and malignant human breast cells. Cell Cycle 2012; 11: 1008–1013. 22333593 10.4161/cc.11.5.19492 PMC3323799

[bib37] McLaughlin-Drubin ME, Park D, Munger K. Tumor suppressor p16INK4A is necessary for survival of cervical carcinoma cell lines. Proc Natl Acad Sci USA 2013; 110: 16175–16180. 24046371 10.1073/pnas.1310432110 PMC3791710

[bib38] Wang Y, Sharpless N, Chang S. p16(INK4a) protects against dysfunctional telomere-induced ATR-dependent DNA damage responses. J Clin Invest 2013; 123: 4489–4501. 24091330 10.1172/JCI69574 PMC3784543

[bib39] Janzen V, Forkert R, Fleming HE, Saito Y, Waring MT, Dombkowski DM et al. Stem-cell ageing modified by the cyclin-dependent kinase inhibitor p16INK4a. Nature 2006; 443: 421–426. 16957735 10.1038/nature05159

[bib40] Krishnamurthy J, Ramsey MR, Ligon KL, Torrice C, Koh A, Bonner-Weir S et al. p16INK4a induces an age-dependent decline in islet regenerative potential. Nature 2006; 443: 453–457. 16957737 10.1038/nature05092

[bib41] Molofsky AV, Slutsky SG, Joseph NM, He S, Pardal R, Krishnamurthy J et al. Increasing p16INK4a expression decreases forebrain progenitors and neurogenesis during ageing. Nature 2006; 443: 448–452. 16957738 10.1038/nature05091 PMC2586960

[bib42] Rayess H, Wang MB, Srivatsan ES. Cellular senescence and tumor suppressor gene p16. Int J Cancer 2012; 130: 1715–1725. 22025288 10.1002/ijc.27316 PMC3288293

[bib43] Lazaro S, Perez-Crespo M, Belen Enguita A, Hernandez P, Martinez-Palacio J, Oteo M et al. Ablating all three retinoblastoma family members in mouse lung leads to neuroendocrine tumor formation. Oncotarget 2016; 8: 4373–4386. 10.18632/oncotarget.13875 PMC5354839 27966456

[bib44] Nana-Sinkam SP, Powell CA. Molecular Biology of Lung Cancer: Diagnosis and Management of Lung Cancer, 3rd ed: American College of Chest Physicians Evidence-Based Clinical Practice Guidelines. Chest 2013; 143: e30S–e309. 23649444 10.1378/chest.12-2346 PMC3961820

[bib45] Schmitt MW, Prindle MJ, Loeb LA. Implications of genetic heterogeneity in cancer. Ann N Y Acad Sci 2012; 1267: 110–116. 22954224 10.1111/j.1749-6632.2012.06590.x PMC3674777

[bib46] Kim D-H, Nelson HH, Wiencke JK, Zheng S, Christiani DC, Wain JC et al. p16INK4a and Histology-specific Methylation of CpG Islands by Exposure to Tobacco Smoke in Non-Small Cell Lung Cancer. Cancer Res 2001; 61: 3419–3424. 11309302

[bib47] Sutherland Kate D, Proost N, Brouns I, Adriaensen D, Song J-Y, Berns A. Cell of origin of small cell lung cancer: inactivation of Trp53 and Rb1 in distinct cell types of adult mouse lung. Cancer Cell 2011; 19: 754–764. 21665149 10.1016/j.ccr.2011.04.019

[bib48] Sutherland KD, Song J-Y, Kwon MC, Proost N, Zevenhoven J, Berns A. Multiple cells-of-origin of mutant K-Ras–induced mouse lung adenocarcinoma. Proc Natl Acad Sci USA 2014; 111: 4952–4957. 24586047 10.1073/pnas.1319963111 PMC3977239

[bib49] Rice WR, Conkright JJ, Na CL, Ikegami M, Shannon JM, Weaver TE. Maintenance of the mouse type II cell phenotype *in vitro*. Am J Physiol Lung Cell Mol Physiol 2002; 283: L256–L264. 12114186 10.1152/ajplung.00302.2001

[bib50] Todaro GJ, Green H. Quantitative studies of the growth of mouse embryo cells in culture and their development into established lines. J Cell Biol 1963; 17: 299–313. 13985244 10.1083/jcb.17.2.299 PMC2106200

