# Supplementary figures and images for: p16 controls epithelial cell growth and suppresses carcinogenesis through mechanisms that do not require RB1 function
Source: Oncogenesis. 2017 Apr 17;6(4):e320–. doi: 10.1038/oncsis.2017.5 (PMC5520502; doi:10.1038/oncsis.2017.5)

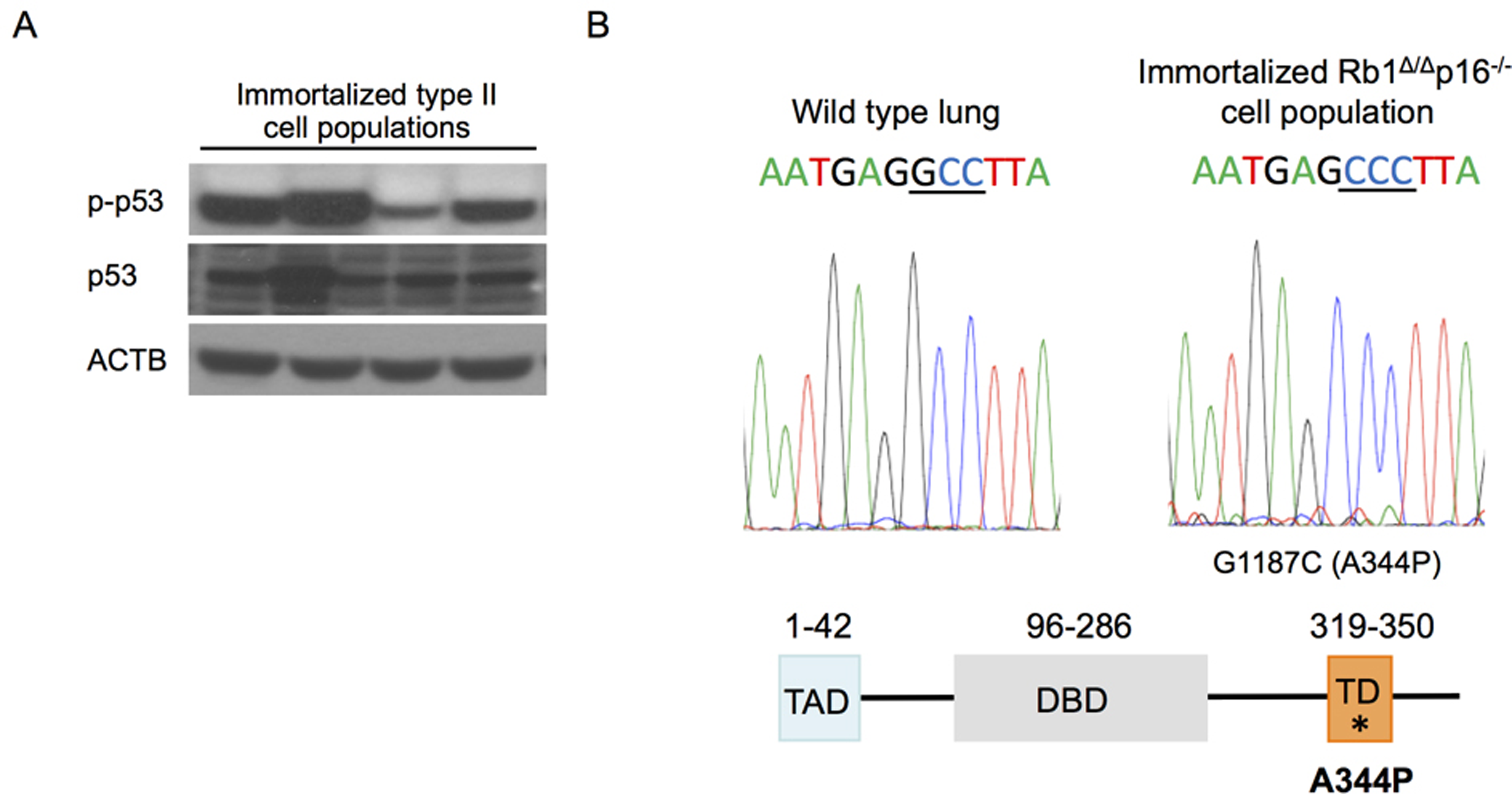

Supplement: Supplementary Figure 1 [file oncsis20175x1.tif]
