# Supplementary material for: p16 controls epithelial cell growth and suppresses carcinogenesis through mechanisms that do not require RB1 function
Source: Oncogenesis. 2017 Apr 17;6(4):e320–. doi: 10.1038/oncsis.2017.5 (PMC5520502; doi:10.1038/oncsis.2017.5)
Supplement: Supplementary Information [file oncsis20175x2.pdf]

SUPPLEMENTAL MATERIAL

Supplemental Fig. TP53 is expressed in all independently derived immortalized Rb1/p16 deficient cell populations with TP53 mutation detected in a single cell population. (A) Activated TP53 phosphorylated at serine 15 (p-p53) and total TP53 (p53) protein was expressed in all 23 immortalized type II cell populations analyzed by western blot analysis with four representative populations shown. Lysates were evenly loaded as assessed by reprobing for ACTB. (B) Exon sequencing of TP53 identified a point mutation in 1 of 24 independently derived cell populations. The detected TP53 exon 10 missense mutation G1187C results in an A344P (A347P in humans) amino acid change in the tetramerization domain (TD) rather than the more frequent occurrence of hotspot mutations in the DNA binding domain (DBD) or transactivation domain (TAD) in human tumors.

SUPPLEMENTAL TABLE

Table: Primers used for TP53 exon sequencing

	Forward primer (5’-3’)	Reverse primer (5’-3’)
Exon 1	ATGGCGACTATCCAGCTTTG	AGAGGTCTCGTCACGCTCAT
Exon 2	AAGCCATAGGGGTTTGTTTG	GATACAGGTATGGCGGGATG
Exon 3	CCAGCCTGGGATAAGTGAGA	GTCCATGCAGTGAGGTGATG
Exon 4	CAGAGCAGAAAGGGACTTGG	GCATTGAAAGGTCACACGAA
Exon 5	CGACCTCCGTTCTCTCTCCT	CCAGTCCTAACCCCACAGG
Exon 6	GCTCCGATGGTGATGGTAAG	GACGCACAAACCAAAACAAA
Exon 7	GTAGGGAGCGACTTCACCTG	CAGAAGCTGGGGAAGAAACA
Exon 8	TGCTGGTCCTTTTCTTGTCC	GTGACTTTGGGGTGAAGCTC
Exon 9	TTGAGCTTCACCCCAAAGTC	ATGCGAGAGACAGAGGCAAT
Exon 10	TGTCCAGTGCTTCCATCTCA	GGAGGGAGGTCTGGGTAGAG
Exon 11	CCTCCCCTTTTCTGTCTTCC	GGGGCCCAGACAGCTTAT
Exon 3-4	ACTTCCTCCAGAAGATATCC	CGTGCACATAACAGACTTCC
Exon 5-6	TACTCTCCTCCCCTCAATAA	CTCGGGTGGCTCATAAGGTA
Exon 7-8	GCCGGCTCTGAGTATACCACC	CCCGTCCCAGAAGGTTCCCA
Exon 8-9	TGGGAACCTTCTGGGACGGG	CTTGAGGGTGAAATACTCTC
Exon 3, #2	ATATATCCTG GTAAGGCCCA	
Exon 4, #2	CGAGTGTCAG GAGCTCCTGC	
Intron 4, #1		ACGTCCCCTG GAGAGATGCA
